# Unique Features of High-Density Lipoproteins in the Japanese: In Population and in Genetic Factors

**DOI:** 10.3390/nu7042359

**Published:** 2015-04-02

**Authors:** Shinji Yokoyama

**Affiliations:** Nutritional Health Science Research Centre and Bioscience and Biotechnology, Chubu University, Kasugai 487-8501, Japan; E-Mail: syokoyam@isc.chubu.ac.jp; Tel./Fax: +81-568-51-9698

**Keywords:** keyword, HDL, atherosclerosis, Japan, CETP, schistosoma, cholesterol

## Abstract

Despite its gradual increase in the past several decades, the prevalence of atherosclerotic vascular disease is low in Japan. This is largely attributed to difference in lifestyle, especially food and dietary habits, and it may be reflected in certain clinical parameters. Plasma high-density lipoprotein (HDL) levels, a strong counter risk for atherosclerosis, are indeed high among the Japanese. Accordingly, lower HDL seems to contribute more to the development of coronary heart disease (CHD) than an increase in non-HDL lipoproteins at a population level in Japan. Interestingly, average HDL levels in Japan have increased further in the past two decades, and are markedly higher than in Western populations. The reasons and consequences for public health of this increase are still unknown. Simulation for the efficacy of raising HDL cholesterol predicts a decrease in CHD of 70% in Japan, greater than the extent by reducing low-density lipoprotein cholesterol predicted by simulation or achieved in a statin trial. On the other hand, a substantial portion of hyperalphalipoproteinemic population in Japan is accounted for by genetic deficiency of cholesteryl ester transfer protein (CETP), which is also commonly unique in East Asian populations. It is still controversial whether CETP mutations are antiatherogenic. Hepatic Schistosomiasis is proposed as a potential screening factor for historic accumulation of CETP deficiency in East Asia.

## 1. Introduction

Coronary heart disease (CHD) is one of the leading causes of death in most of the industrialized countries of the world. While it is still the number one cause of death in North America and Europe, its prevalence in Japan is one-third or less of that in the Western world in spite of its heavy industrialization, even after the gradual increase in CHD in Japan in the decades post World War II [1]. The major reasons for this difference are largely considered to be environmental factors such as the differences in Japanese life style. That is supported by such findings that Japanese immigrants to the United States show a similar risk of CHD to other Americans [2,3].

In addition, plasma high-density lipoprotein (HDL) concentration, often represented by its cholesterol and a strong epidemiological protective factor against atherosclerotic vascular diseases, is high among the Japanese. It is therefore important to investigate whether this contributes to the lower prevalence of atherosclerotic vascular diseases in Japan, and what environmental and/or genetic factors are responsible for this high HDL. Because of this very population background, low HDL cholesterol can be a stronger risk factor for CHD in Japan than is elevation of non-HDL cholesterol (cholesterol in all other lipoproteins than HDL) [4,5]. This article reviews some unique findings recently unveiled about HDL in the Japanese; its recent remarkable increase in comparison to other parts of the world [6], strong contribution of HDL to CHD risk and therefore its high potential as a target of risk reduction treatment [7], and the particular genetic backgrounds for high HDL in East Asia, including Japan [8,9,10].

## 2. HDL Levels of Japanese

Measurement of HDL cholesterol has been a standard clinical parameter since early 1980s. The technology for its determination has been well developed and established in the past twenty years [11], so that the HDL cholesterol assay is now adequately standardized and the values are reasonably reliable for international comparisons [12,13]. HDL cholesterol has been shown to be a strong inverse predictor of risk for atherosclerosis in almost all epidemiological and interventional studies worldwide [14,15,16,17]. This is also the case in Japan based on many studies, including those of ourselves [3,4], and the prevalence of CHDs is still substantially lower in Japan than Western countries.

**Figure 1:**
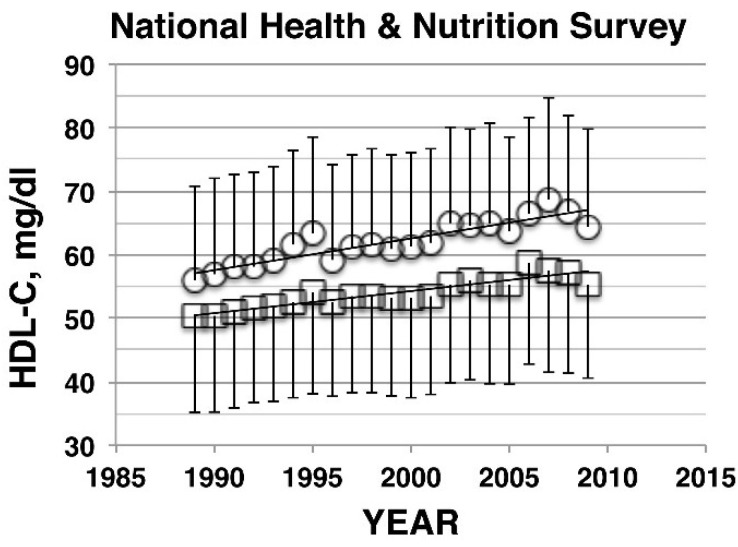
HDL-cholesterol (HDL-C) data in the National Health and Nutrition Survey, mean ± SD, for male (squares) and female (circles). The slopes are 0.35 mg/dL/year and 0.5 mg/dL/year for male and female, respectively, with *p* <0.001 for both [6].

Plasma lipoprotein profiles have been determined in National Health and Nutrition Survey (NHNS), a nationwide survey conducted in Japan by the Ministry of Health, Welfare and Labor, in which HDL cholesterol data are available since 1988 [1]. As demonstrated in Figure 1, a trend of prominent and continuous increase in HDL cholesterol has been seen for the time period of 1988 to 2010 [6]. Linear regression of the plot yielded the slope of the increase to be 0.35 and 0.50 mg/dL/year for both men and women (*p* < 0.001). These values stayed the same even after the subjects taking lipid-related medications were excluded. The same trend was also confirmed in HDL cholesterol data obtained from one of the major commercial clinical laboratories in Japan, SRL, as well as many other cohort studies throughout Japan during this period [6].

On the other hand, the United States National Health and Nutritional Examination Survey demonstrated no significant change in HDL cholesterol for the period of 1966 to 2002 [18], but significant increase between 1988–1994 and 2007–2010 [19]. The increase was significant in both sexes and the total population, but differences were apparent among different ethnic groups; significant changes were only seen in “non-Hispanic white” and this seemed a main factor to cause overall significant increase in the total population. Significant increase was also seen in “non-Hispanic black women” group when the subgroup taking lipid-lowering drugs was excluded. The increase of HDL cholesterol was 4.9% and 5.6% for men and women, respectively, of the non-Hispanic white group and 4.3% and 5.0% for men and women of the entire population surveyed, respectively [19] (The copyright policy of AMA does not allow reusing part of the Tables/Figures from AMA publication to create a new table, so that the readers are advised to refer directly to the reference).

The Japanese population data were analyzed in the same manner to compare with the results of The United States and summarized in Table 1. Both NHNS and SRL data showed the percentage increase of 7%–10% and by 14% for men and women, respectively, between the comparable time periods, more than double the increase in The United States. HDL cholesterol values were higher in Japan than The United States already in the 1988–1994 period by 12.3% and 6.3% in men and women, respectively, of the entire population and 14% and 5.7% in non-Hispanic white men and women, respectively, and the gaps were widened to 19.1% and 14.2% for the former and 20.4% and 13.1% in the latter by the 2010s.

**Table 1:** Change of HDL and non-HDL in mg/dL, Mean ± SE, in the National Health and Nutrition Survey (NHNS) and SRL data.

			1988–1994		2007–2010		% Change	*p* value
HDL-Cholesterol	NHNS	Men	51.3	±14.9	56.8	±15.4	10.6	<0.001
		Women	59.0	±15.1	66.6	±15.5	14.2	<0.001
	SRL	Men	49.4	±15.5	53.1	±14.2	7.3	<0.001
		Women	56.1	±15.7	63.9	±15.7	13.9	<0.001
Apolipoprotein A-I	SRL	Men	123.6	±30.1	134.9	±29.0	9.1	0.01
		Women	133.2	±28.0	151.0	±29.3	13.4	0.02
non HDL-Cholesterol	NHNS	Men	148		142		−4.0	
		Women	145		140		−3.2	

**Figure 2:**
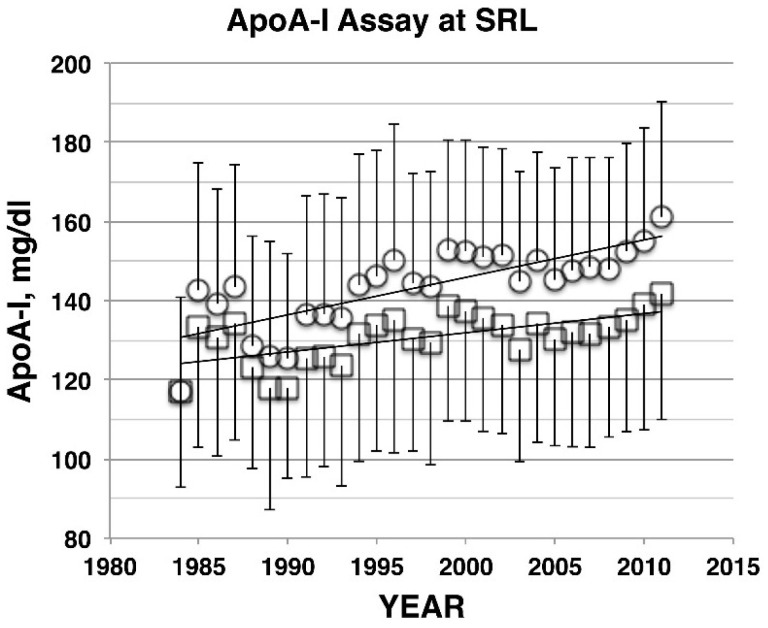
Apolipoprotein A-I (ApoA-I) measured commercially in the SRL laboratory, mean ± SD, for male (squares) and female (circles). The slopes are 0.485 mg/dL/year and 1.064 mg/dL/year for male and female, respectively, with *p* < 0.001 for both [6].

The possibility remained that this trend was caused by technical errors, such as drifting of standardization for the assay systems, as the methodology for HDL cholesterol measurement has been shifted during the periods the surveys covered, from precipitation of apoB lipoproteins to homogeneous and automated assay systems. In order to exclude the possibility of such artifacts, independent parameters for HDL have been explored. Apolipoprotein A-I (apoA-I) has been measured in SRL by using an enzyme-linked immune assay system, which followed international standardization and stayed with the same technology throughout the period. As demonstrated in Figure 2 and Table 1, apoA-I data obtained from SRL showed a similar trend of increase to that observed with HDL cholesterol [6]. The increase was also confirmed in the data obtained in the Serum Lipid Survey by the Research Group for Primary Hyperlipidemia under the Ministry of Health, Welfare and Labor of Japan, conducted in 1990 and 2000 independently of NHNS [6,20,21]. HDL cholesterol increased in all age groups of both men and women by 10.3% and 12.6%, respectively, being consistent with other results. In contrast, non-HDL cholesterol values decreased in the both countries, but seemed more in the United States than in Japan [6,19]. The age distribution profile of non-HDL cholesterol was almost superimposable between 1990 and 2000 in Serum Lipid Survey [6,20,21].

We thus conclude that the increase of HDL is very likely a real phenomenon, both in the United States and Japan. Average HDL level among Japanese is apparently higher than that of Americans of most ethnic groups. It was already higher 25 years ago and the difference became much greater in the past 20 years, reaching almost 20% in men and 13%–14% in women (Table 1).

## 3. Simulation of the Effect of Raising HDL on CHD in Japan

It is an important question to what extent the change in plasma concentration of each lipoprotein subfraction would contribute to the risk of atherosclerotic vascular diseases in the Japanese population. The prevalence of stroke and CHD is roughly similar in Japan but lipoprotein risks for the former are not apparently detected, perhaps because the aging risk masks them. On the other hand, many cohort or observational trial studies in Japan, including those of ourselves, demonstrated that plasma HDL cholesterol is the strongest risk predictor for CHD among lipoprotein-related parameters, including low density lipoprotein (LDL) cholesterol [4,5]. Pharmacological reduction of LDL-cholesterol did demonstrate reduction of the risk of CHD by some 30% in Japan even though LDL is not the leading risk factor [22]. Therefore, it is not irrational to conceive that HDL cholesterol could be a better target to prevent CHD, assuming that raising HDL reverses the risk similarly to reduction of LDL. We therefore attempted to conduct such a simulation [7].

The age-adjusted prevalence of myocardial infarction was obtained as events per year per population of 1000 for every segment of serum LDL-cholesterol and HDL-cholesterol concentration, from the cohort study Japan Lipid Intervention Trial (J-LIT), six-year follow-up study of 47,294 patients treated with low-dose simvastatin, carried out between 1992 and 1999 [23]. Serum lipoprotein cholesterol concentration profiles among Japanese were taken from the study by the Research Committee on Serum Lipid Levels Survey 1990 in Japan [20]. Population statistics of Japan were taken from the Census Japan 1995 [24] for the age group of 50–70 to match the age range of the subjects monitored in J-LIT and population profiles of serum LDL-cholesterol and HDL-cholesterol were calculated for this age group. We concluded that raising HDL cholesterol is more efficient than reduction of LDL cholesterol for prevention of myocardial infarction (MI) in Japan based on the assumption that the former approach is valid to reverse the risk, although, sex difference has not been taken into consideration [7].

**Figure 3:**
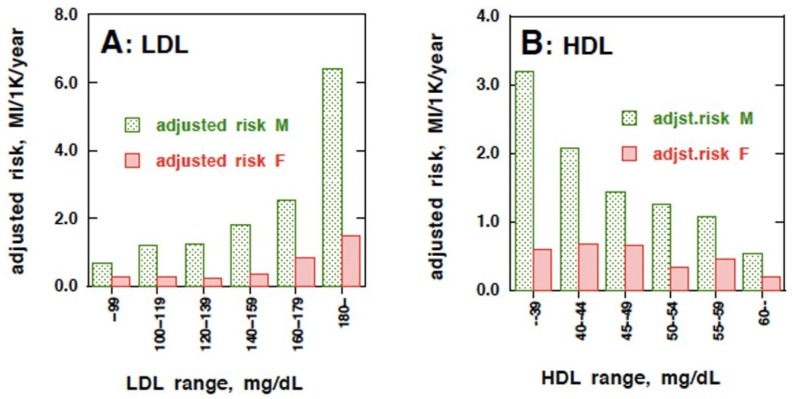
Age-adjusted risk of myocardial infarction (MI) dependent of plasma lipoprotein-cholesterol for each sex. The risk is expressed as events per 1000 population per year (MI/1K/1Y) in J-LIT trial [25].

As the further detail of J-LIT data became available for male and female subjects separately [25] (Figure 3), the same simulation was conducted for each sex for this review. The treatment efficacy was empirically calculated by the method previously described [7]. The process of the calculation can be followed as the data in Table 2 for male. The population in each lipoprotein concentration sextile was determined from its distribution statistics [20]. The number of new patients a year was predicted for each segment by using the primary risk estimates of MI for each lipoprotein segment group in J-LIT study [23,25]. Reduction of MI in the population segments by reducing LDL to the next lower segment was estimated by the difference of the primary risk between these segments, assuming that the risk is reversible by reducing LDL concentration. This calculation was sequentially performed to simulate reduction of the numbers of the patients by reducing LDL of the entire population from one segment to the next towards the lowest LDL segment. For HDL, the same procedure was applied, except that the calculation starts with the lowest HDL group to shift to the higher segment. The efficacy of the treatment (assuming it is “effective”) was calculated as the number needed to treat (NNT) for each level of lipoprotein target.

The numbers of new MI patients a year were predicted for each segment group of LDL or HDL in Table 2 from the population in each LDL or HDL concentration segment group and the risk-adjusted MI prevalence in the J-LIT study as MI/1000/year (MI/K/Y). The numbers in each line of LDL/HDL segment (I–VI) in Table 2 indicate MI/K/Y when the people in the respective segment are treated to reduce LDL cholesterol (2A) or increase HDL cholesterol (2B) to the segment (I–VI) represented by each column. Therefore, the sum of each column (the bottom line, total) indicates MI/K/Y when the segment level of the column was reached by the treatment of the entire population. For example, the population with LDL above 180 mg/dL is 854,000 and the number of MI is predicted as 5449 per year (Table 2A). If the LDL level of this entire population group can be reduced to the 160–180 mg/dL segment, the number of MI in this original group decreases to 2178, and then it becomes 1554 when LDL is reduced further to the 140–160 mg/dL segment. A similar assumption was followed for the effect of raising HDL in Table 2B. Parameters for the “treatment efficacy” were calculated as NNT in Table 3. Overall decreases of the MI patients by LDL reduction/HDL elevation were taken from the values in Table 2. The population needed to treat to accomplish the particular lipoprotein level segment was calculated (to reach the 120–140 mg/dL of LDL cholesterol segment, add 854K, 2196K and 3660K in Table 2A, for example). The NNT can be thus calculated for each LDL target segment. For graphical illustration of NNT for each sextile as the efficacy of the treatment, the inverse of the value (×1000) was used.

**Table 2:** (**A**) Decrease of myocardial infarction (MI) by reducing LDL cholesterol (LDL-C; male), according to the method in ref. [7]; (**B**) Decrease of MI by increasing HDL cholesterol (HDL-C; male).

(A)										
Segment	LDL-C		Population	MI/K/Y	Number of MI/Y when the Segment Targeted					
	mg/dL		(K)		VI	V	IV	III	II	I
I		−100	6100	0.67	4087	4087	4087	4087	4087	4087
II	100	−120	5856	1.18	6910	6910	6910	6910	6910	3924
III	120	−140	5734	1.22	6995	6995	6995	6995	6776	3842
IV	140	−160	3660	1.82	6661	6661	6661	4465	4319	2452
V	160	−180	2196	1.82	5600	5600	3997	2679	2591	1471
VI	180	-	854	6.38	5449	2178	1554	1042	1008	572
Total			24,400		35,702	32,431	30,204	26,178	25,691	16,348
(**B**)										
**Segment**	**HDL-C**		**Population**	**MI/K/Y**	**Number of MI/Y when the Segment Targeted**					
	**mg/dL**		**(K)**		**VI**	**V**	**IV**	**III**	**II**	**I**
I	60	-	5612	0.53	2974	2974	2974	2974	2974	2974
II	55	−60	3172	1.08	3426	3426	3426	3426	3426	1681
III	50	−55	3416	1.25	4270	4270	4270	4270	3689	1810
IV	45	−50	3904	1.43	5583	5583	5583	4880	4216	2069
V	40	−45	3172	2.07	6566	6566	4536	3965	3426	1681
VI		−40	5124	3.2	16397	10607	7327	6405	5534	2716
Total			24,400		39,216	33,426	28,116	25,920	23,265	12,931

Each line represent the group of the subjects of lipoprotein cholesterol level (LDL-C or HDL-C) defined as I to VI with population of the age between 50 and 70 (J-LIT age) and risk of myocardial infarction (MI/K/Y), and predicted numbers when lipoprotein-C is managed towards the target segment as VI to I. Total indicates the sum of the column, *i.e.*, the numbers of MI when each lipoprotein-C target is achieved. When no treatment is done, the number of MI is predicted as 35,702 while it is down to 30,204 if the goal of the segment IV is achieved for the entire population.

**Table 3:** (**A**) Number needed to treat (NNT) in prevention of myocardial infarction (MI) by increasing LDL cholesterol (male); (**B**) NNT in prevention of MI by increasing HDL cholesterol (male).

(A)						
HDL Segment Targeted	I	II	III	IV	V	VI
	(–100)	(100–120)	(120–140)	(140–160)	(160–180)	(180–)
Total MI	16,348	25,691	26,178	30,204	32,431	35,702
% reduction	46	72	73	85	91	100
MI Overall Decreased	19,354	10,011	9527	5498	3271	
Population to Treat (K)	18,300	12,444	6710	3050	854	
NNT	946	1242	704	555	261	
1/NNT × 1000	1.5	0.805	1.42	1.8	3.83	
(**B**)						
**HDL Segment Targeted**	**I**	**II**	**III**	**IV**	**V**	**VI**
	**(60–)**	**(55–60)**	**(50–55)**	**(45–50)**	**(40–45)**	**(–40)**
Total MI	12,931	23,265	25,920	28,116	33,426	39,216
% reduction	33	59	66	72	85	100
MI Overall Decreased	26,285	15,951	13,296	11,100	5790	
Population to Treat (K)	18,788	15,616	12,200	8296	5125	
NNT	714	979	918	737	885	
1/NNT × 1000	1.401	1.021	1.089	1.451	1.134	

Parameters for treatment efficacy as each lipoprotein cholesterol target (I to VI) is achieved, based on the date in Table 2. NNT: number needed to treat ((decreased MI)/(Population to treat)). Calculation was, according to the method in ref. [7].

As demonstrated in Table 3, the estimated reduction of MI prevalence using this simulation is greater by raising HDL than reducing LDL in Japanese male subjects aged 50 to 70, assuming that the risk is reversible based on changes in the plasma concentration of each lipoprotein.

**Figure 4:**
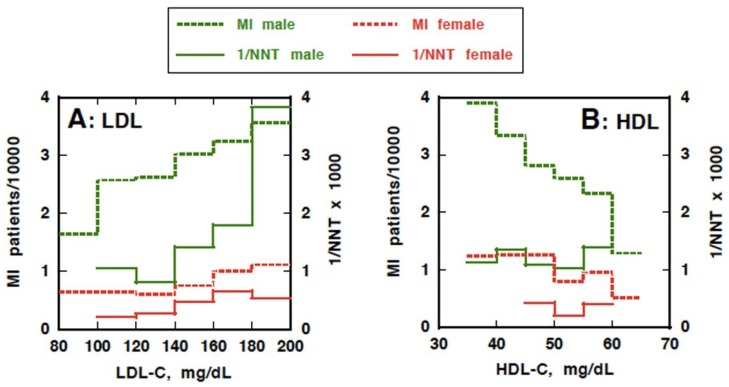
Prevention of ischemic heart disease in Japanese by decreasing LDL-cholesterol (LDL-C) and increasing HDL-cholesterol (HDL-C). Simulation for prevention of myocardial infarction (MI) based on the graphs in Figure 3 and demographic data of Japanese, for males and females, according to the data represented in Table 2 and Table 3. Solid lines represent the inverse of NNT (×1000) as an indicator of the treatment efficacy for managing lipoproteins to a target. The value of each horizontal segment is the efficacy when reaching a target LDL or HDL level at the left or right end of the segment, respectively, in all Japanese at ages covered by the JLIT. Each horizontal segment of broken lines represents the number of MI patients when LDL is decreased or HDL is raised to the left end of the segment.

Figure 4 represents a graphical view of the results of the simulation for decrease of MI by reduction of LDL (4A) and raising HDL (4B) both in males and females. The data shown are for the number of patients per year as the lipoprotein treatment target is achieved for all those to be treated, and for NNT to reach this goal, calculated for each lipoprotein target levels (the values for male are listed in Table 3). For LDL reduction in males, the treatment efficacy (1/NNT) sharply dropped when the treatment goal is lowered from 180 to 160 mg/dL, and then gradually decreased to the level that NNT is a little higher than 1000. The final potential prevention of MI is estimated at about 50% by reducing LDL to 100 mg/dL. In females, the same tendency was demonstrated but with much lower incidence of CHD, but the data might not be qualified for conclusive analysis, since the number of the events was so low.

In the simulation of increasing HDL, the treatment efficacy did not diminish as much as the lowering of LDL when HDL cholesterol increased from below 40 mg/dL to over 60 mg/dL. The NNT stayed at around the level somewhat less than 1000 throughout the range of change in HDL. The reduction of MI is linear and would reach approximately 30% of the original level by raising HDL cholesterol to the segment over 60 mg/dL. In females, the results were essentially similar to those in males but not highly conclusive because of low incidence of events.

Our simulation model predicted that MI among Japanese could be prevented by at most 50% by aggressive reduction of LDL, but could reach as much as 70% by interventions raising HDL, granted it is feasible. LDL reduction, however, may have an advantage over raising HDL with respect to the treatment effectiveness (NNT) as far as setting a target higher than 160 mg/dL. These results indicate that elevation of LDL is not as great a risk factor for CHD in the Japanese as is low HDL, and low HDL should be considered as a more important target to reduce the overall risk.

## 4. A Genetic Factor that Increases HDL among the Japanese

Plasma HDL concentration, typically represented by HDL cholesterol content, is likely more important for Japanese public health than in the Western world. This is suggested by the higher average HDL concentration in Japan, the fact that CHD events are lower in Japan, and based on the simulation model presented here predicting the potential of more efficient reduction of atherosclerotic cardiac diseases by raising HDL than by reducing LDL. It is quite conceivable that some genetic factors may also contribute to the higher plasma HDL concentration in Japan. One genetic factor resulting in higher HDL cholesterol and higher frequency in Japan are dysfunctional mutations of cholesteryl ester transfer protein (CETP).

CETP is a glycosilated plasma protein with a molecular weight of 74,000 that catalyzes the non-directional equimolar exchange of non-polar lipids, mainly cholesteryl acylester (CE) and triglyceride (TG), among lipoprotein particles core [26,27,28]. CETP is present in the plasma of some animals, such as rabbits, hamsters and some primates including humans [29]. The reaction equalizes distribution of the core lipids among lipoproteins. CE is actively generated in plasma HDL by the lecithin: cholesterol acyltransferase (LCAT) reaction and TG originates in secretion of very-low-density lipoproteins (VLDL) and chylomicrons by the liver and the intestinal cells, respectively. Accordingly, CETP mediates the net movement of CE from HDL to VLDL/chylomicrons and TG from VLDL/chylomicron to HDL and low-density lipoproteins (LDL) [26], and consequently regulates the HDL cholesterol level in plasma [30]. Increase of the plasma TG therefore results in decrease of HDL cholesterol, which may partially explain the cardiovascular risk of hypertriglyceridemia. In turn, HDL-CE increases when the CETP reaction decreases. Generation of CE by LCAT in HDL is one of the driving forces for the removal of cell cholesterol [31], and is an important part of cholesterol transport from somatic cells to its catabolic site, the liver. The CETP reaction may facilitate this transport by sending CE from HDL to the LDL pathway for removal from plasma through the hepatic LDL receptor [29].

The patients with CETP deficiency patients were first discovered in Japan in 1985 in individuals with extremely high HDL cholesterol [32,33] and CETP mutations were subsequently identified [34]. This was found to be a frequent cause of hyperalphalipoproteinemia among Japanese. The two major mutations account for the majority of the cases are intron 14 G(+1)-to-A (Int14A) and exon 15 missense mutation (D442G) [35,36,37]. These two mutants are present in 1% to 2% and 6% to 7% of the Japanese population, respectively. Sporadic cases of other CETP mutations have also been identified among Japanese [36,37,38,39,40]. Accordingly, the estimated numbers of CETP mutation heterozygotes in japan is around 10 million, with 150,000 to 250,000 having mutations in both CETP alleles. Based on this frequency, CETP deficiency may account for 27.6% of the people with HDL cholesterol ≥60 mg/dL and 31.4%–32.5% of those with HDL ≥80 mg/dL in Japan [35,41].

The first non-Japanese patient with CETP deficiency was of Chinese descent, reported from Switzerland [42]. Several reports thereafter described CETP deficiency among other Asians. The prevalence of CETP D442G was found to be 2.1%–3.8% in the Mainland Chinese population [43,44], and 4.5%–6.7% in the population of Taiwan [45,46]. The prevalence of D442G was estimated to be 12% among Koreans based on an allele frequency of 6% [47] and 6.9% in the Vietnamese population [48]. D442G heterozygosity was found to be the cause of nine out of the 35 individuals with hyperalphalipoproteinemias in a study in Thailand [49], accounting for 26% of cases, similar to the ratio among the Japanese [35,41] and strongly indicating that CETP mutants are common in Thailand. Further detailed information is available in the previous review article [50]. More recent result for Siberian Yakuts indicates the prevalence of D442G mutant is 16.3% in the native Yakuts, compared to 5.2% among the non-indigenous Siberians [51], whose intermarriage rate with Yakuts is estimated at 10% to 20% (Ariev AL, personal communication). Not much reliable information is available for the Int14A CETP mutation, except for two out of the 145 subjects (1.4%) in Hong Kong Chinese [52] and none of 346 Vietnamese [48]. In the Omagari of Akita district in Northern Japan, accumulation of Int14A has a heterozygote prevalence of 27% [37]. These up-to-date data are summarized in Table 4.

**Table 4:** Allele Frequency identified as prevalence of mutants, summarized by Thompson *et al*. * [50], unless otherwise referred to **.

	D442G	In14	Number
			Genotyped
Japanese Americans	5.1	0.49	3469
Japanese not on meds	8.1	0.60	2267
Osaka controls	6.0	1.00	514
Japanese children	6.0	0.00	500
Japanese hemodialysis	6.5	--	414
Japanese FH	3.5	0.69	288
Japanese centenarians	6.3	0.78	256
Japanese controls	6.8	1.69	236
Japanese normal HDL	13.7	4.42	226
Japanese controls	6.8	1.58	190
Ohmagari controls	4.0	27.0	173
Chinese controls	4.2	--	379
Chinese controls	5.0	1.00	335
Chinese controls	3.3	--	209
Chinese CHD	10.8	--	203
Chinese CHD	3.5	0.00	200
Chinese stroke	3.6	0.91	110
Chinese Healthy elderly	3.0	--	103
Chinese MI	3.5	1.05	94
Hong Kong Chinese **	--	1.4	145
			(Thu *et al.* [48])
Taiwanese controls	6.7	718	
Taiwanese controls	4.7	--	278
Taiwanese controls	4.5	--	224
Taiwanese CHD	7.7	--	196
Korean **	12	--	270
			(Song *et al*. [47])
Korean post menopausal	9.2	--	228
Vietnamese	6.9	0.00	
Yakuts **	16.3	--	144
			(Arkhipova *et al*. [51])
Russians/Ukrainians **	5.2	--	116
(10%–20% cross-marriage with Yakuts)			(Arkhipova *et al*. [51])
North Indian controls	0.0	0.0	
French healthy controls	0.0				
Scottish case/controls	0.0		
Caucasians **	(<1.0) (Thompson *et al.* [50])		

In contrast, genetic CETP deficiency is rare in ethnic groups other than East Asians. No D442G mutations were found among the 400 individuals examined in North India [53]. The first Caucasian case was reported in 1997 [54], and the presence of one case of Int14A was recognized in 1998 in Canada without ethnic background information [55]. It was concluded that CETP deficiency is rare among North American Caucasians [56]. Nevertheless, a few studies reported sporadic cases of CETP deficiency from the United States [57], Italy [58,59], and the Netherlands [56,60]. There was a statement for the frequency of D442G mutant as “less than 1%” in US without providing the data [61].

In summary, CETP deficiency is highly prevalent in East Asia, at least among the Japanese, Chinese both in Mainland and Taiwan, and Koreans, predominantly with the D442G mutant. It is likely be similarly as frequent in Thailand, Vietnam and Siberian native people. Int14A mutant may be a second common mutation, but it is not clearly evident except in Japan. Many other less-frequent types of mutation have also been found in Japan, but such information is not available so far from other Asian regions. In contrast, it is rare in any other ethnic groups in other parts of the world. The two common Asian mutations are not commonly identified even among the sporadic cases from other continents. Thus, geographic or ethnic distribution of CETP mutations is extremely unique. It can be hypothesized that there is a unique environmental reason(s) for this peculiar accumulation of CETP mutants, other than Omagari, Japan, that is potentially due to a founders’ effect [37].

Clinical manifestations of CETP deficiency are limited to the abnormal plasma lipoprotein profile, including markedly elevated HDL cholesterol and moderately reduced LDL cholesterol. With reduced CETP activity, CE generated by LCAT is retained in HDL while its substrate molecules of free cholesterol and glycerophospholipids are continuously supplied to HDL from other lipoproteins and cells, including erythrocytes by non-specific exchange or other transfer reactions [31]. Thus, HDL particles become larger as the core CE compartment expands. Therefore, the apparent increase of HDL cholesterol is not due to an increase in HDL particle number but because of the enlargement of the particles, to the extent of reaching diameters as large as LDL [62,63,64]. LDL particles in turn get somewhat smaller and contain an increased amount of TG in their core and resulting in overall reduction of LDL-cholesterol [65,66]. No obvious clinical symptoms are present in CETP-deficient individuals.

**Figure 5:**
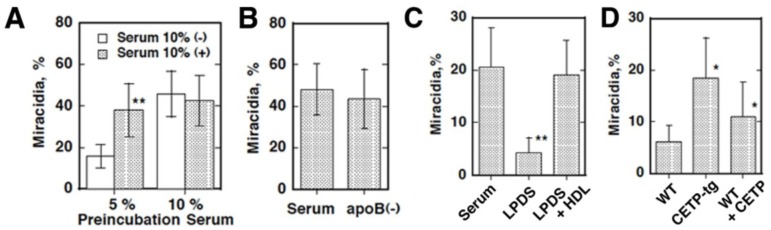

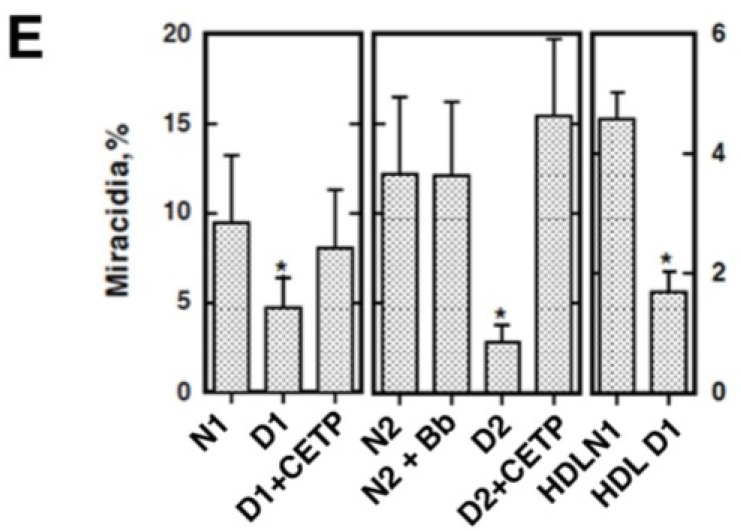
Lipoprotein requirement for embryonation of the *S. japonicum* eggs after eight days in culture [7,8,9,10]. (**A**): incubation of the eggs with and without human serum (10%) after pre-exposure of the parents while laying the eggs to 5% and 10% of human serum. Embryonation proceeds when the parents or eggs have access to adequate human serum. Preincubation with 5% serum is applied hereafter; (**B**): Embryonation is not influenced even when apoB-lipoprotein is removed from human serum (apoB (-)); (**C**): Embyonation requires the HDL fraction of human serum. LPDS; lipoprotein deficient serum; (**D**): The effect of mouse serum on the egg embryonation. Embryonation is poor with wild-type mouse serum that lacks CETP and proceeds with CETP-transgenic mouse serum (CETP-tg). Adding human CETP to the wild-type serum partially restored embryonation; (**E**): Embryonation of the *S. japonicum* eggs after eight days in culture with CETP-deficient human serum, taken from the reference [8]. Embryonation is estimated in the culture of the eggs in 10% normal human sera (N1 and N2) and that of CETP-deficient subjects (D1 and D2). Embryonation is retarded in CETP-deficient serum (**A**) and adding CETP recovers this (**B**). Normal HDL is adequate for the embryonation but not HDL from CETP-deficiency (**C**). ** *p* < 0.01 and * *p* < 0.05 from serum (-) (**A**), serum (**C**), WT (**D**), normal HDL (**E**).

**Figure 6:**
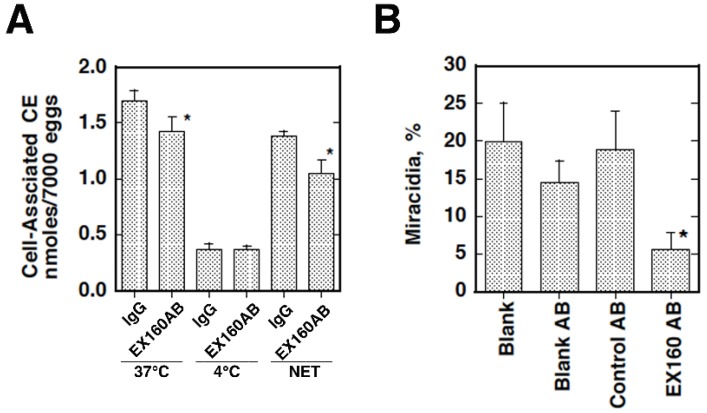
Suppression of cholesteryl acylester (CE) uptake **(A)** and embryonation of the *S. japonicum* eggs **(B)** by the antibody raised against the extracellular domain peptide representing the residues 249–408 of CD36RP. Blank AB, nonimmunized rabbit antibody, Control AB, antibody against the intracellular domain of CD36RP (residues 331–348) [9,10].

**Figure 7:**
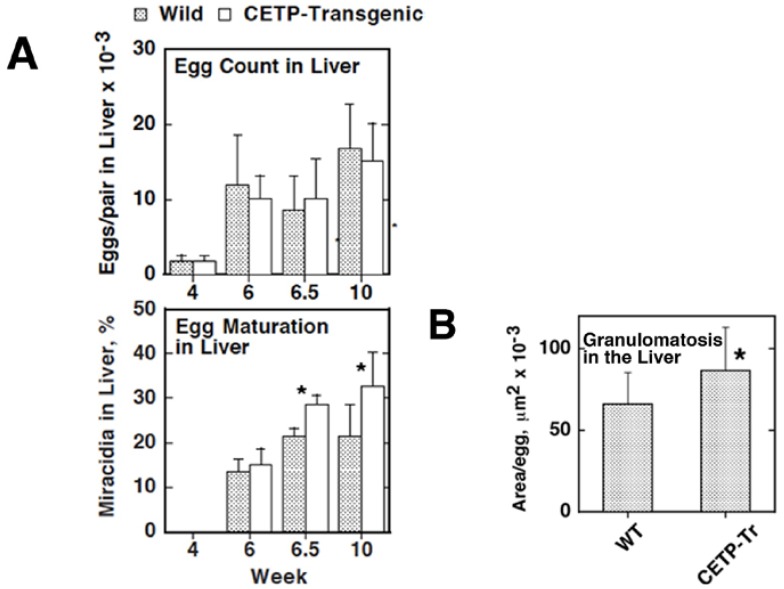
Egg embryonation and the liver lesion development in the wild-type and CETP-transgenic mice infected with *S. japonicum*. (**A**): Number of the eggs embolizing in the liver and ectopic embryonation of the eggs counted microscopically; (**B**): Granulomatosis lesion in the liver. The eggs and granulomatous lesions were identified microscopically in the liver specimens. The total area of the lesion per egg was calculated for each section. The area per egg was calculated as an average ± SE of the 12 mice, for each of which 12 random liver sections were examined. Asterisks indicate difference at *p* < 0.05 from the wild type [8,10].

We thus investigated region-specific and potentially fatal diseases that might be associated with plasma lipoprotein metabolism to explore a screening factor for accumulation of CETP mutations in East Asia, and focused on *Schistosoma japonicum*, an Asian-specific blood fluke [8,9,10]. Parasitic blood flukes reside as a pair in the host portal and intestinal veins and use plasma lipoproteins as nutrient sources through the lipoprotein receptor-like systems. A portion of the eggs laid for excretion through the gastrointestinal tract go into the liver via the portal circulation and undergo ectopic embryonation to cause hepatic granulomatosis and fatal cirrhosis. Accordingly, we examined *Schistosoma japonicum*, with respect to dependency of its pathogenesis on plasma lipoproteins. The parasites were shown to take up CE from HDL for the embryonation of their eggs to miracidia, which is a critical step of the hepatic pathogenesis of this parasite, but this reaction was shown to be impaired from the HDL of CETP-deficiency (Figure 5). Due to similarity of the reaction to those by scavenger receptor B1 or CLA-1 of selective CE uptake from HDL in rodents or humans, we searched for a factor responsible for this reaction as a CD-36 family protein(s). A new protein, CD36-related protein (CD36RP), was cloned from the adults and the eggs of *Schistosoma japonicum*, with 1880-bp encoding 506 amino-acid residues exhibiting the CD36 domains and two transmembrane regions. The extracellular domain of this protein selectively bound normal human HDL but neither LDL nor CETP-deficiency HDL, and the antibody against the extracellular domain suppressed the selective HDL-CE uptake from normal HDL and embryonation of the eggs (Figure 6). When infected with *Schistosoma japonicum*, wild-type mice developed less hepatic granulomatosis than CETP-transgenic mice by the ectopic egg embryonation (Figure 7). CD36RP is thus a candidate receptor of *Schistosoma japonicum* to facilitate uptake of HDL-CE necessary for egg embryonation. Abnormal HDL caused by CETP-deficiency retards this process and thereby protects the patients from development of hepatic lesions in this infection.

## 5. Discussion

Plasma HDL levels of the Japanese were shown to be high based on HDL cholesterol content and apoA-I levels, and have gradually but substantially increased even further during the past 20 years to reach the levels 15%–20% higher than the average in the United States (Table 1) [6,19]. This may contribute at least in part to low incidence of CHD, including myocardial infarction in japan [4,5]. Conversely, low HDL is a strong epidemiological risk for CHD in Japan. Raising HDL (cholesterol, at least in simulations) may potentially prevent the CHD more efficiently than reduction of LDL, assuming that correcting plasma lipoprotein concentrations can reverse the risk (Table 3, Figure 4) [7]. One of the genetic causes for high HDL in Japan is a high prevalence of mutation in the CETP gene, which is uniquely common in East Asia, at least from the Indochina Peninsula to Siberia. The two major mutations are present in 6%–10% of general population and may account for 25%–30% of high HDL populations in these regions. Homozygotes of CETP deficiency seem to be resistant to development of fatal hepatic Schistosomiasis providing survival advantage for development of these mutations in this region [8,9,10]. Further analysis will be required to determine whether the increase of HDL over time or the specific genetic background, *i.e.*, CETP mutation, is responsible for low prevalence of CHD in Japan.

There is no doubt that CETP deficiency contributes to the average high HDL concentration in Japan [35,40]. However, it is unknown whether the increase of abnormal HDL caused by CETP mutations plays an anti-atherogenic role as suggested by the reduced CHD with high HDL in population studies. HDL particles become as large as LDL with substantial accumulation of apolipoprotein E in homozygous deficiency of CETP [67]. It has been wondered whether CETP deficiency is protective against atherosclerotic vascular diseases, and the answer has been controversial and inconclusive in epidemiological cohort studies on heterozygotes [37,68,69]. Abnormal HDLs present in the homozygotes may not be as anti-atherogenic as normal HDL, since they show apparently impaired capacity for removal of cell cholesterol [70]; in addition, the homozygotes patients do not show resistance to atherosclerosis [37].

Pharmacological inhibition of CETP has been attempted in the past decade, and has demonstrated very positive results in a rabbit model of diet-induced hyperlipoproteinemia [68]. However, clinical trials have so far failed to prove the positive effect of CETP inhibitors [71,72]. The trials are ethically obliged to demonstrate additional effect of raising HDL on a background of statin treatment so that a part of the beneficial effect of raising HDL may have been masked. Furthermore, the doses of statins used in these trials were relatively high because the subjects of the trials were those at high risk. On the other hand, it is argued that inhibition of CETP may not be beneficial as it blocks the routing cholesteryl esters from HDL to LDL/VLDL for its more efficient flow to the liver and causes accumulation of cholesteryl ester in HDL. In addition, consequent enlargement of the particle may diminish the ability of HDL to remove cell cholesterol [70]. It is thus inconclusive whether inhibition of CETP is beneficial in spite of it being an efficient way to increase HDL. When considering these backgrounds, we cannot answer the question yet whether high prevalence of CETP deficiency contributes to low incidence of CHD in Japan. Alternatively, CETP inhibitors can potentially be useful to prevent development of fatal hepatic schistosomiasis.

An increase in average HDL levels over the past 20 years has occurred both in Japan and in the United States [6,19] (Table 1). The magnitude of the increase was much greater in Japan and it made the gap in HDL levels between the two countries much wider. It is conceivable that extensive health education and an intentional trend in lifestyle changes were major factors to lead to such results in both countries. An increase of average HDL cholesterol by around 5% of general population in the United States is significant when compared to the increase of HDL in many clinical trials using statins or even fibrates. A decrease in plasma triglycerides is associated with increases in HDL cholesterol as the CETP reaction causes reciprocal changes of these parameters [26,30], and decreases of triglyceride were indeed observed in the same survey [19]. However, such a relationship is not maintained throughout different ethnic groups in the United States, so that this is unlikely the major reason for HDL cholesterol increase [19]. As the change is more prominent in Adults than youths in the United States, lifestyle change seems more likely the cause [73].

The magnitude of increase in HDL cholesterol in Japan for the same period of time was almost 10% to 15%, much greater than that in the United States. Changes in plasma triglycerides were not significant at all during this period [1], so that the reason for this change is presumed to be other factors related to the environment, including lifestyle change.

Most of the fundamental changes in lifestyle in Japan took place during the immediate postwar time, 1946–1975, along with rapid economic growth, drastic shifting in the energy source of power and great changes in the fundamentals of society, such as large increase in car and telephone ownership and changing food quantity and quality. Total calorie intake increased by 20% with the increase of protein by 15% and fat by 3.5 fold, and the decrease in carbohydrate by 15% from the peak intake in 1955. These changes all must have pushed plasma lipid parameters towards an atherogenic direction. However, such trends were more or less stabilized after the mid seventies, and total caloric intake gradually decreased by 20% after the peak time of 1977, along with further substantial decreases in carbohydrate intake by 23%. In the same period of time, alcoholic consumption remained the same, smoking rates decreased in males but increased a little in females, and intentional exercise may have increased a bit. The most remarkable change in lifestyle in Japan is perhaps as anywhere else, the rapid spread of intellectual technology, and lives became greatly dependent on the use of cell phones and the Internet. However, by any of those or even altogether, it is difficult to explain the increase of HDL by 10% to 15%.

Interesting changes are found in Japanese food intake by looking into detail at trends after World War II (NHNS, [1]). While seafood intake has shown only mild increase from 65 to 90 g/capita/day by 1975 and slightly declined to 70 g/capita/day again after 2000, sharp increases were seen in the intake of animal meat products from 5 to 75 g/capita/day by 1980 and reaching 80 g/capita/day in 1990s, milk products from 1 to 100 g/capita/day by 1975, hitting a peak of 140 g/capita/day in 1995 and declining thereafter to 120 g/capita/day, and egg and egg products from 2 to 40 g/capita/day by 1975. These nutritional changes were apparently one of the driving forces for extending average lifespan of the Japanese to one of the highest in the world. According to the United Nations Food and Agricultural Organization (UNFAO) [74], Japan is one of the top consumers of fish per capita, along with Portugal, Norway and Korea. However, the ratios of seafood to meat in calorie intake per capita basis are 0.16, 0.29 and 0.45, respectively, in these countries while that of Japan was 0.99 in 2010. The ratios were 0.16, 0.13 and 0.08 in France, Italy and the United States, respectively. Thus, even after such a tremendous change in food intake pattern, Japanese are still the highest fish eaters in the world.

**Figure 8:**
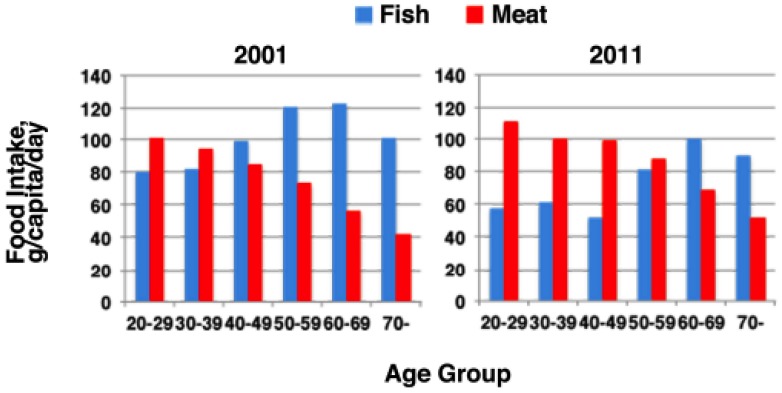
Source of food intake by the age groups of Japanese, according to the National Health and Nutrition Survey [1].

This background, however, may not be likely to account for the increase of HDL in the past 20 years unless the drastic change in the time more immediately after the war had some distant or prolonged effects. On the other hand, a more recent important trend in food intake of Japanese is found in NHNS data (Figure 8). From 2001 to 2011, the ratio of fish intake to meat may have only slightly decreased for the overall population mainly by shrinking fish consumption. However, the ratio dropped to almost half in the age groups from 20 to 50 during these 10 years. It will be very important to monitor this trend in relation with various risk factors for lifestyle-related diseases, including HDL-related parameters and CHD incidence.

## 6. Conclusions

The situations related to HDL are thus quite unique in Japan in comparison to other part of the world, with respect to both environmental and genetic factors of Japanese, in association with long historic backgrounds. The questions have to be set to address whether any of these factors may be involved in the lower incidence of CHD in Japan. The attempt to draw answers even partially should contribute to solving worldwide public health problems of prevention and cure of atherosclerotic vascular diseases. There is no direct evidence yet, however, that these unique findings are associated with any public health problem of Japan. Extensive investigation is required.
